# Rabbit hemorrhagic disease.

**DOI:** 10.3201/eid0402.980237

**Published:** 1998

**Authors:** C Mead


calicivirus. Actually, RHDV is one of the best characterized caliciviruses, and the publication of its full genome sequence in 1991 was the first of a *Caliciviridae* member (5).

Diagnostic tools have been developed by our and other laboratories (3,4,6). Thanks also to specific monoclonal antibodies produced towards RHDV and European brown hare syndrome virus (EBHSV) by our colleague E. Brocchi, we standardized different enzyme-linked immunosorbent assays (ELISAs) for the diagnosis of related diseases (4,6-8). In particular, we developed five different ELISAs for serology that allow the detection of antibodies specific for RHDV or EBHSV or that are cross-reactive. In addition, we can define the antibody response in rabbits and hares in terms of isotype-involved immunoglobulin M (IgM), IgA, and IgG (9). Today the main difficulty is the qualitative distinction between RHDV and rabbit calicivirus (RCV, a recently identified nonpathogenic calicivirus) antibodies because of the close antigenic profiles of these viruses (6). Finally, RHDV- and EBHSV-specific polymerase chain reaction has been developed in at least five laboratories besides ours. We have sent these reagents and/or diagnostic methods to at least 19 laboratories outside Italy, including Australia, New Zealand, and the United States.

Does RHDV infect humans? This question has arisen together with the prospect of using RHDV as a biologic control agent in countries like Australia and New Zealand, when they were free of RHDV. In Europe, where the disease naturally occurred and quickly spread, no particular control on human health was planned. In Italy only, between 1987 and 1990, hundreds of millions of rabbits died of RHD in regions where the average density of humans is very high. As a consequence of the use of the vaccine since 1991, the incidence of RHD among breeding rabbits decreased drastically and quickly. Nevertheless, the disease is still endemic, mainly in small farms and among wild rabbits. EBHS also is endemic in wild hares, and hunters are highly exposed to the virus since hares are their main target. However, neither in humans nor in animal species other than rabbits and hares have any diseases similar to RHD ever been reported. In relation to the likelihood of mild or inapparent infections, we used 100 human sera randomly selected from blood donors to carry out a preliminary standardization of an RHD-ELISA that has been periodically used to control the sera of the RHD laboratory staff. Very recently, we tested nine sera from laboratory personnel exposed to RHDV; again no positive result was noted by RHD-ELISA. These findings have limited epidemiologic value, but considering the high level of exposure of part of the sample, it is evident that RHDV infection in humans is unlikely to be the rule.

**Lorenzo Capucci and Antonio Lavazza**
Istituto Zooprofilattico Sperimentale della Lombardia e dell'Emilia, Brescia, Italy

### References

1. Murphy FA. Virus taxonomy. In: Fields BN, Knipe DM, Howley PM, editors. Virology. 3rd ed. New York: Lippincott-Raven Publishers; 1996. p. 15-57.
2. Ohlinger VF, Hass B, Meyers G, Weiland F, Thiel HJ. Identification and characterization of the virus causing rabbit hemorrhagic disease. J Virol 1990;64:3331-6.
3. Rodák L, Šmíd B, Valícek L, Veselý T, Stepánek J, Hampl J, et al. Enzyme-linked immunosorbent assay of antibodies to rabbit haemorrhagic disease virus and determination of its major structural proteins. J Gen Virol 1990;71:1075-80.
4. Capucci L, Scicluna MT, Lavazza A. Diagnosis of viral hemorrhagic disease of rabbits and European brown hare syndrome. Rev Sci Tech 1991;10:347-70.
5. Meyers G, Wirblich C, Thiel HJ. Rabbit hemorrhagic disease virus-molecular cloning and nucleotide sequencing of a calicivirus genome. Virol 1991;184:664-76.
6. Lavazza A, Capucci L. Viral haemorrhagic disease of rabbits. In: Office International des Epizooties, Paris, France, Manual of standards for diagnostic test and vaccine. Paris: the Office; 1996. p. 589-98.
7. Capucci L, Fusi P, Lavazza A, Pacciarini ML, Rossi C. Detection and preliminary characterization of a new rabbit calicivirus related to hemorrhagic disease virus but nonpathogenic. J Virol 1996;70;8614-23.
8. Capucci L, Frigoli G, Ronsholt L, Lavazza A, Brocchi E, Rossi C. Antigenicity of the rabbit hemorrhagic disease virus studied by its reactivity with monoclonal antibodies. Virus Res 1995;37:221-38.
9. Capucci L, Nardin A, Lavazza A. Seroconversion in an industrial unit of rabbits infected with a non-pathogenic rabbit haemorrhagic disease-like virus. Vet Rec 1997;140:647-50.

### Rabbit Hemorrhagic Disease

**To the Editor**: The recent article on calicivirus by Smith et al. (1) is misleading in its use of the study concerning human health aspects of rabbit hemorrhagic disease (RHD) by Mead et al. (2).

The RHD exposure categories of "low" and "high" used by Mead et al. and mentioned in the first column of page 18 (1) are not related to the categories of "low" and "high" given in the same paragraph at the top of the second column. The reader might easily assume that it was Mead et al. who considered that Jul–Dec 1995 was "a low exposure period." This is not so—such a classification is made by Smith et al.

Further, the reader might assume that it was the study by Mead et al. that concluded "that exposure to RHD virus remains a plausible explanation for increased disease incidence." Again this is an inference drawn by Smith et al. and is the opposite of the conclusion of Mead et al.

The basis of exposure in the study by Mead et al. is at an individual level—the respondents were chosen either because they had been handling rabbits or as controls in determining the level of disease. In contrast, Smith et al. consider exposure at a broad environmental level and disregard whether the respondents had been handling infected rabbits or not. Actually, more contact with rabbits occurred during the first half of the study than during the second.

Smith et al. do not mention the conclusions of Mead et al.: These neither showed any significant difference between levels or types of illness in those exposed and those not exposed to RHD virus nor demonstrated any association between the exposure to RHD and number of episodes of illness in the subsequent 1 to 2 months.

The results of the study by Mead et al. may be summarized by noting that the average number of episodes of illness over the 13-month reporting period was 2.6 for respondents who had not been exposed to RHD virus, 2.2 for those classified as having a low level of exposure, and 2.3 for those classified as having a high level.

The study by Mead et al. concluded that, on the basis of the health survey and the lack of any serologic reaction of the respondents, there was considerable support to the view that RHD virus is not associated with infection or disease in humans. The results of the study have been submitted for publication in a scientific journal.

Reference 31 should refer to the Bureau of Resource Sciences (not Studies).

**C. Mead**
Convenor, Rabbit Calicivirus Human Health Study Group, Department of Health and Family Services, Canberra, Australia

### References

1. Smith AW, Skilling DE, Cherry N, Mead JH, Matson DO. Calicivirus emergence from ocean reservoirs: zoonotic and interspecies movements. Emerg Infect Dis 1998;4:13-20.
2. Mead C, Kaldor J, Canton M, Gamer G, Crerar S, Thomas S. Rabbit calicivirus and human health. Canberra, Australia: Department of Primary Industries and Energy, Australian Government (Released under the Official Information Act). Report of the Rabbit Calicivirus Human Health Study Group; 1996.

### Reply to Drs. Capucci, Lavazza, and Mead

**To the Editor:** We are aware of Capucci and Lavazza's excellent work. Indeed, one of the best characterized calicivirus genomes is that detected in rabbit hemorrhagic disease (RHD); however, the virus' infectivity, pathogenesis, modes of transmission, reservoirs, survival in nature, host of origin, virulence factors, number of neutralization serotypes, and multispecies infectivity are poorly characterized. Propagating this virus in vitro could provide insight for addressing questions relevant to caliciviruses that cannot be propagated in vitro.

We are unclear about the confusion regarding Norwalk virus and feline calicivirus (FCV). Both are caliciviruses. Norwalk virus is a human pathogen. FCV is in a different genus (1) that includes strains infecting humans (2). We know of no documented FCV infections in humans nor of detailed studies to search for such occurrences, although some evidence suggests the possibility (3).

Capucci and Lavazza's remaining questions address the etiology of RHD, diagnostic reagents, and possible human infection. They report nine laboratory workers as antibody negative but do not report test results on persons at high risk, such as rabbit farm workers, nor do they mention having positive control human or primate sera. Koch's postulates have been fulfilled for RHD: a parvovirus was isolated in vitro and was cell-passaged 15 times; at a second laboratory, the parvovirus was identified in materials causing RHD (4,5). In Europe the parvovirus etiology for RHD was deemed hypothetical but has not been refuted on a scientific basis. The calicivirus consistently identified in European materials has not been isolated in vitro, and Koch's postulates have not been fulfilled. Are the parvovirus-associated outbreaks of RHD in Mexico and China (4,5) and the calicivirus-associated RHD

